# *Pinb-D1p* is an elite allele for improving end-use quality in wheat (*Triticum aestivum* L.)

**DOI:** 10.1007/s00122-022-04232-7

**Published:** 2022-09-29

**Authors:** Siyuan Chang, Qian Chen, Tao Yang, Binyong Li, Mingming Xin, Zhenqi Su, Jinkun Du, Weilong Guo, Zhaorong Hu, Jie Liu, Huiru Peng, Zhongfu Ni, Qixin Sun, Yingyin Yao

**Affiliations:** State Key Laboratory for Agrobiotechnology, Frontiers Science Center for Molecular Design Breeding, Key Laboratory of Crop Heterosis and Utilization (MOE), and Beijing Key Laboratory of Crop Genetic Improvement, China Agricultural University, Beijing, 100193 China

## Abstract

**Key message:**

We identified ten QTLs controlling SDS-SV trait in a RIL population derived from ND3331 and Zang1817. *Pinb-D1p* is an elite allele from Tibetan semi‑wild wheat for good end-use quality.

**Abstract:**

Gluten strength is an important factor for wheat processing and end-product quality and is commonly characterized using the sodium dodecyl sulfate-sedimentation volume (SDS-SV) test. The objective of this study was to identify quantitative trait loci (QTLs) associated with wheat SDS-SV traits using a recombinant inbred line (RIL) population derived from common wheat line NongDa3331 (ND3331) and Tibetan semi-wild wheat accession Zang1817. We detected 10 QTLs controlling SDS-SV on chromosomes 1A, 1B, 3A, 4A, 4B, 5A, 5D, 6B and 7A, with individual QTLs explaining 2.02% to 15.53% of the phenotypic variation. They included four major QTLs, *Qsdss-1A*, *Qsdss-1B.1*, *Qsdss-1B.2*, and *Qsdss-5D*, whose effects on SDS-SV were due to the *Glu-A1* locus encoding the high-molecular-weight glutenin subunit 1Ax1, the 1B/1R translocation, 1Bx7 + 1By8 at the *Glu-B1* locus, and the hardness-controlling loci *Pina-D1* and *Pinb-D1*, respectively. We developed KASP markers for the *Glu-A1*, *Glu-B1*, and *Pinb-D1* loci. Importantly, we showed for the first time that the hardness allele *Pinb-D1p* positively affects SDS-SV, making it a good candidate for wheat quality improvement. These results broaden our understanding of the genetic characterization of SDS-SV, and the QTLs identified are potential target regions for fine-mapping and marker-assisted selection in wheat breeding programs.

**Supplementary Information:**

The online version contains supplementary material available at 10.1007/s00122-022-04232-7.

## Introduction

Bread wheat (*Triticum aestivum* L.) is one of the most widely cultivated crops in the world and a major source of plant-based protein in the human diet, providing 20% of the calories and protein in the global human diet (Shiferaw et al. 2013). Wheat flour has widespread uses in staple foods, such as baked and steamed breads, noodles, cakes, and cookies; therefore, end-use quality improvement is a major aim in wheat breeding programs. Gluten, composed of the seed storage proteins glutenin and gliadin, provides the unique extensibility and elasticity of dough and is a major contributor to end-use quality of wheat flour (Balakireva and Zamyatnin 2016). Parameters associated with gluten content and quality, such as the sodium dodecyl sulfate-sedimentation volume (SDS-SV), wet gluten content, gluten index, dough stability time, and dough tensile resistance, are widely used to qualify the end-use quality of wheat flour. However, most of these are difficult to assess due to the complicated and time-consuming testing procedures and the requirement for many homozygous seeds, which are often not available, particularly in the early selection stage of wheat breeding when the amount of seeds per plant or progeny is low. The SDS-SV test offers advantages such as simplicity, low cost, a small sample size requirement, and high efficiency. This test has been widely used in wheat breeding programs to discriminate wheat genotypes showing good end-use quality (Carter et al. 1999). The SDS-SV is associated with the content and strength of gluten and is well correlated with baking quality of wheat flour (Axford et al. 1979). For example, mutant wheat lines lacking the high-molecular-weight glutenin subunit (HMW-GS) loci *Glu-A1*, *Glu-B1*, or *Glu-D1* showed significantly decreased glutenin content and SDS-SV compared with wild-type Xiaoyan 81 (Yang et al. 2014). A lack of HMW-GS 1Bx7 or 1By9 leads to a lower SDS-SV and inferior sponge cake quality (Chen et al. 2019). The SDS-SV is also positively correlated with other quality-related parameters, including protein content, gluten index, mixograph mixing time, extension/extensibility ratio, and farinograph parameters such as development time, stability time, and water absorption (Dexter and Matsuo 1980; Dhaka and Khatkar 2015; Tian et al. 2021). Overall, the SDS-SV trait is an important quality parameter that can be utilized in wheat quality evaluation.

The SDS-SV trait of wheat is generally controlled by multiple genes, and inherited as quantitative trait loci (QTLs). The phenotypes of QTL-controlled traits are often influenced by environmental factors and exhibit high genotype–environment interactions (Zhang et al. 2008). QTLs for SDS-SV have been identified on almost all chromosomes by genome-wide QTL mapping in different populations. However, the major QTLs associated with this trait are mainly associated with multiple genes encoding glutenins and gliadins, such as the HMW-GS loci *Glu-1* (*Glu-A1*, *Glu-B1*, and *Glu-D1*); low-molecular-weight glutenin loci *Glu-A3* and *Glu-B3*; and the gliadin locus *Gli-B1* (Chen et al. 2020; Deng et al. 2015; Guo et al. 2020b; Semagn et al. 2021; Würschum et al. 2016). For example, two QTLs, *QSds.dms-1A* and *QSds.dms-1B*, were found to explain 41% and 19% of the variation in SDS-SV. *QSds.dms-1B* was mapped to position 96 cM on chromosome 1B, which is the expected location of *Glu-B1*, and *QSds.dms-1A* was mapped to a region harboring a cluster of genes including *Glu-A3* and *Gli-A3* (Semagn et al. 2021). In addition, a putative novel QTL for SDS-SV, *QSsv.cau-1A.1.1* (371.6–386.4 Mb, IWGSC RefSeqv1.0), was mapped to chromosome 1A, where it does not overlap either *Glu-A1* (508.7 Mb, IWGSC RefSeqv1.0) or *Glu-A3* (4.2 Mb, IWGSC RefSeqv1.0); this QTL explained 17.21–26.47% of the phenotypic variation in SDS-SV (Tian et al. 2021). Thus, further fine-mapping and map-based cloning of new QTLs for the SDS-SV trait are still needed to clarify the locations of the associated genes.

Puroindolines constitute about 5–10% of the protein fraction in wheat grain (Morris 2002) and are the major determinant of grain hardness, which in turn affects milling and end-use quality. Puroindolines are encoded by the *Puroindoline a* (*Pina-D1*) and *Puroindoline b* (*Pinb-D1*) genes, located at the *Hardness* locus (*Ha*) on chromosome 5D. Wheat cultivars with wild-type *Pina-D1a* and *Pinb-D1a* genotypes have soft-textured seeds, whereas mutations in either *Pina-D1a* or *Pinb-D1a* lead to hard-textured seeds (Bhave and Morris 2008). Previous studies have reported eight allelic variants at the *Pina-D1* locus (*Pina-D1b*, *f*, *k–n*, *p*, and *q*) and 17 allelic variants at the *Pinb-D1* locus, including 13 types of nucleotide mutation (*Pinb-D1b-g*, *l*, *q*, *t*, *v*, *w*, *aa*, and *ab*) and four frameshift mutations (*Pinb-D1p*, *r*, *s*, and *u*) (Bhave and Morris 2008). Among the puroindoline mutations, the *Pinb-D1b* allele has a positive effect on the SDS-SV (Würschum et al. 2016). However, the effects of other hardness alleles on wheat quality are still unclear.

Tibetan semi-wild wheat (*Triticum aestivum* ssp. *tibetanum* Shao) provides a significant resource for wheat genetic improvement and is adapted to the environmental extremes of high-altitude conditions (Guo et al. 2020a). Some elite QTLs associated with yield-related traits, including plant height, spike length, spikes per plant, grain weight per spike, thousand-grain weight, and flag leaf angle, have been identified from Tibetan semi-wild wheat accession Zang1817 (Liu et al. 2014, 2018). As high-altitude conditions trigger extensive reshaping of wheat genomes, the potential contribution of Tibetan semi-wild wheat to wheat quality improvement remains to be investigated. The objectives of the present study were to i) map QTLs associated with SDS-SV using a recombinant inbred line (RIL) mapping population derived from two wheat cultivars, low SDS-SV parent ‘ND3331’ and high SDS-SV ‘Zang1817’, and ii) develop molecular markers linked to QTLs for wheat quality breeding. We identified four major QTLs associated with SDS-SV and found that the hardness allele *Pinb-D1p* is a good candidate for SDS-SV trait improvement. The results of this study provide a better understanding of the genetic mechanisms controlling the SDS-SV trait and show that Tibetan landraces might be helpful for the genetic improvement of wheat quality.

## Materials and methods

### Plant materials and field trials

A total of 189 F_10_ RILs derived from a cross between common wheat line NongDa3331 (ND3331) and a Tibetan semi-wild wheat Zang1817 using the single-seed descent method were used for QTL mapping of the SDS-SV trait. The RILs and parents were grown in Qingdao (36°N, 120°E), Shandong province, in 2019 (2019-QD) and 2021 (2021-QD) and in Handan (36°N, 114°E), Hebei province, in 2020 (2020-HD) and 2021 (2021-HD). In each environment, the RILs and parents were planted in a randomized complete block with three replications. Each replication contained two rows 1.5 m long and 0.3 m apart, sowed at a rate of 20 seeds per row. All field trials were well watered and managed in accordance with local standard practices.

### SDS-SV testing

Grain samples harvested from the field were stored for approximately 2 months, adjusted to 14% moisture content, and then milled using a Perten 3100 experimental mill to obtain whole flour. Two grams of the whole wheat flour sample were then used for SDS-SV tests according to a previously published method (Dick and Quick 1983) with some modifications (2 g of flour, sedimentation volume was recorded after 5 min). The flour was suspended with bromophenol blue solution (1%). Protein hydration was facilitated by the addition of sodium dodecyl sulfate, which is a mild detergent, and lactic acid. The SDS-SV (mL) was determined as the volume below the interface line between the solid (ground sample) and liquid (solution) in a measuring cylinder. Three biological replicates were analyzed for each sample.

### Linkage map construction and QTL analysis

Genomic DNA of parents and individual RILs was extracted from fresh seedling leaves using the cetyltrimethylammonium bromide (CTAB) method (Webb and Knapp 1990), and DNA quality and quantity were assessed using a NanoDrop ND-1000 spectrophotometer (Thermo Scientific, Wilmington, DE, USA). The 55 K wheat single-nucleotide polymorphism (SNP) Genotyping Array (China Golden Marker Co., Beijing, China) was used for genotyping. The filtering criteria for SNPs for linkage map construction were as follows: SNPs showed polymorphisms between two parents were retained, then the polymorphic SNPs with > 10% missing genotype information and the redundant and co-segregation SNPs with the same genetic distance were removed, the remaining SNPs were used to construct genetic linkage map. Map distances were converted from recombination frequencies using the Kosambi mapping function (Kosambi 1944), and JoinMap4.0 and Mapchart v2.32 software were used to create the genetic linkage map (Ooijen et al. 2006; Voorrips 2002). Averaged trait values for plants grown in each environment were used for QTL analysis. Windows QTL Cartographer v2.5 software was used for composite interval mapping (CIM) to identify and analyze QTLs (Wang et al. 2005). Limit-of-detection (LOD) scores were calculated with 1,000 permutations at *P* ≤ 0.05, and a QTL with LOD ≥ 2.5 was defined as significant. The *R*^2^ value was estimated as the percentage of variance explained by each locus in proportion to the total phenotypic variance.

### Genome resequencing and InDel marker development

High-quality genomic DNA of ND3331 and Zang1817 was extracted and sequenced with an average 6 × coverage of the assembled genome using the Illumina NovaSeq 6000 platform as 2 × 150 bp reads. High-quality reads were aligned to the Chinese Spring IWGSC RefSeq v1.0 using the Burrows-Wheeler Aligner 0.7.15 program with default parameters (Li and Durbin 2009). Insertion/deletion (InDel) calling was performed using the HaplotypeCaller module, and InDels between Nongda3331 and Zang1817 were used to develop molecular markers for QTL analysis (Dataset S1). Primer 3 was used to design the sequences of InDel primers.

DNA amplification was programmed for an initial 5 min at 94 °C, followed by 35 cycles of 30 s at 94 °C, 30 s at 58 °C, and 30 s at 72 °C, and finally 5 min at 72 °C. A 10 µL PCR reaction system was used, containing 5 µL of 2 × Taq PCR StarMix (GenStar, Beijing, China), 1.5 µL of DNA template (about 50–100 ng), 1.5 µL of each InDel primer, and double-distilled H_2_O. The PCR products were analyzed on 8% non-denaturing polyacrylamide gels with silver staining.

### Statistical analysis

The skewness and kurtosis were calculated using the “SKEW” and “KURT” functions in IBM SPSS Statistics 20 (SPSS, Chicago, USA). Two-tailed Student’s *t* tests were used to determine differences in parental phenotypes. Broad-sense heritability was calculated using the PROC GLM procedure in SAS (SAS Institute, Cary, NC, USA), based on the following formula: *H*^2^ = *V*_G_∕(*V*_G_ + *V*_GE_ + *V*_E_), where *V*_G_ is genetic variance, *V*_GE_ is the variance of genotype × environment interaction and *V*_E_ is the residual error. Correlations between pairs of the SDS-SV trait in the RIL population among different environments (Dataset S2) were determined using Pearson’s correlation analyses with IBM SPSS Statistics 20 (SPSS, Chicago, USA).

## Results

### SDS-SV varied in the RIL population generated from ND3331 and Zang1817

We used 189 F_10_ RILs derived from a cross between common wheat line NongDa3331 (ND3331) and Tibetan semi-wild wheat accession Zang1817 for QTL mapping of the sodium dodecyl sulfate-sedimentation volume (SDS-SV) trait. The mean SDS-SV of Zang1817 was significantly higher than that of ND3331 in all four environments (Table 1). The broad-sense heritability of SDS-SV was 0.92, indicating that SDS-SV is mainly controlled by genetic factors (Table 1). The SDS-SV exhibited a normal distribution in the RIL population in all four environments, and the frequency distribution showed continuous variation, indicating that this trait is determined by multiple genes (Fig. S1). There was a significant and positive correlation in the values for the SDS-SV trait among all four environments (Table 2).

**Table 1:** Phenotypic performance and distribution of the SDS-SV trait in two parents and RIL populations

Trait	Environment	ND3331	Zang1817	RIL				
		Mean	Mean	Mean ± SD	Range	Skewness	Kurtosis	*H2*
SDS-SV (mL)	2019-QD	13.48	24.82**	17.10 ± 3.95	8.37–27.57	− 0.20	− 0.54	0.92
	2020-HD	12.83	21.30**	15.22 ± 2.31	10.13–20.17	− 0.07	− 0.61	
	2021-QD	10.93	18.73**	13.66 ± 2.55	7.93–19.67	− 0.12	− 0.73	
	2021-HD	11.30	19.53**	14.33 ± 2.52	8.67–21.40	− 0.02	− 0.44	

All the data is the average of the phenotypes in each environment. *RIL* recombinant inbred line. *SDS-SV* sodium dodecyl sulfate-sedimentation volume. *QD* Qingdao, Shandong province; *HD* Handan, Hebei province. Double asterisks indicate significant differences determined by a two-tailed Student’s *t* test at *P* < 0.01

**Table 2:** Pearson’s correlation coefficients of SDS-SV among four environments

Environment	2019-QD	2020-HD	2021-QD
2020-HD	0.837**		
2021-QD	0.743**	0.742**	
2021-HD	0.825**	0.866**	0.831**

SDS-SV, sodium dodecyl sulfate-sedimentation volume. QD, Qingdao, Shandong province; HD, Handan, Hebei province. **Correlation is significant at *P* < 0.01 (two-tailed Student’s *t* test)

### Construction of a genetic linkage map

We used the 55 K wheat single-nucleotide polymorphism (SNP) Genotyping Array (China Golden Marker Co., Beijing, China) containing 53,063 SNPs to analyze the genotype of the RIL population and two parents. A total of 9,838 (18.54%) SNPs showed polymorphism between two parents. After removing the polymorphic SNPs with > 10% missing genotype information and the redundant and co-segregation SNPs with the same genetic distance, 1,174 SNPs were used to construct the genetic linkage map; they were mapped to 21 linkage groups, distributed across all 21 chromosomes of common wheat (Table S1). The total length of the map was 5,508.84 cM, with an average spacing of 4.69 cM. The A, B, and D genomes included 451 (38.42%), 419 (35.69%), and 304 (25.89%) markers covering lengths of 1,833.02, 1,970.86, and 1,704.96 cM, with average marker intervals of 4.06, 4.70, and 5.61 cM, respectively (Table S1).

### Alleles from Zang1817 at four stable loci contribute to higher SDS-SV

We identified 10 QTLs with LOD > 2.5 associated with the SDS-SV trait on chromosomes 1A, 1B, 3A, 4A, 4B, 5A, 5D, 6B, and 7A in all four environments (Table 3). Among them, four QTLs, *Qsdss-1A*, *Qsdss-1B.1*, *Qsdss-1B.2*, and *Qsdss-5D*, were stably detected in at least three environments and six (*Qsdss-3A*, *Qsdss-4A*, *Qsdss-4B*, *Qsdss-5A*, *Qsdss-6B*, and *Qsdss-7A*) were detected in one or two environments (Table 3). These 10 QTLs explained 2.02–15.53% of the phenotypic variances in the different environments (Table 3).

**Table 3:** QTLs for the SDS-SV trait detected in all environments in the ND3331 and Zang1817 RIL populations

QTL	Environment	Chromosome	Marker interval	Physical interval (Mb)	LOD	*R*^2^ (%)	*Add*
*Qsdss-1A*	2019-QD	1A	*AX-111601840*—*AX-110430171*	474.7–511.8	9.4	9.21	− 1.23
	2020-HD	1A	*AX-111601840*—*AX-110430171*	474.7–511.8	8.5	7.08	− 0.63
	2021-QD	1A	*AX-111601840*—*AX-110430171*	474.7–511.8	3.3	4.09	− 0.53
	2021-HD	1A	*AX-111601840*—*AX-110430171*	474.7–511.8	6	5.94	− 0.63
*Qsdss-1B.1*	2019-QD	1B	*AX-109503584*—*AX-108726602*	329.7–383.2	10.5	9.60	− 1.54
	2020-HD	1B	*AX-109840997*—*AX-108966369*	1.3–347.6	17.7	15.53	− 1.13
	2021-QD	1B	*AX-109840997*—*AX-108941599*	3.7–347.6	6.7	8.56	− 0.94
	2021-HD	1B	*AX-109840997*—*AX-108941599*	3.7–347.6	8.3	8.54	− 0.92
*Qsdss-1B.2*	2019-QD	1B	*AX-86184300*—*AX-111619113*	555.3–570.8	10.7	9.79	− 1.53
	2020-HD	1B	*AX-86184300*—*AX-110041248*	555.3–564.4	10.2	8.83	− 0.85
	2021-QD	1B	*AX-86184300*—*AX-110409210*	555.3–570.8	8.1	10.21	− 1.01
	2021-HD	1B	*AX-86184300*—*AX-111619113*	555.3–577.7	9.5	9.81	− 0.98
*Qsdss-3A*	2019-QD	3A	*AX-110439700*—*AX-109956297*	580.2–649.4	2.6	2.02	− 0.57
	2020-HD	3A	*AX-110439700*—*AX-109956297*	580.2–649.4	3.4	2.92	− 0.4
*Qsdss-4A*	2019-QD	4A	*AX-109924369*—*AX-110367474*	692.7–735.8	2.9	2.28	− 0.61
*Qsdss-4B*	2021-QD	4B	*AX-109881538*—*AX-110144838*	269.5–541.4	4.2	4.96	0.59
*Qsdss-5A*	2020-HD	5A	*AX-109864419*—*AX-109369408*	459.7–477.1	4	3.19	− 0.42
*Qsdss-5D*	2019-QD	5D	*INDEL1489469*—*INDEL8181683*	1.5–8.2	6.5	5.71	− 0.95
	2020-HD	5D	*INDEL1489469*—*INDEL8181683*	1.5–8.2	8.7	10.80	− 0.76
	2021-HD	5D	*INDEL1489469*—*INDEL8181683*	1.5–8.2	5.2	7.82	− 0.71
*Qsdss-6B*	2021-QD	6B	*AX-111050096*—*AX-110501144*	34.2–147.6	2.5	3.61	0.44
*Qsdss-7A*	2019-QD	7A	*AX-110011761*—*AX-109865315*	56.1–82.2	3.3	2.78	0.66
	2020-HD	7A	*AX-110011761*—*AX-109865315*	56.1–82.2	3.4	2.31	0.35

*RIL* recombinant inbred line. *SDS-SV* sodium dodecyl sulfate-sedimentation volume. *QD* Qingdao, Shandong province; *HD* Handan, Hebei province. Position represents the distance of the peak LOD value from the left marker; physical interval (Mb) was determined according to IWGSC RefSeq v1.0 (http://www.wheatgenome.org/); LOD represents the maximum-likelihood LOD score for the QTLs; *R*^2^ (%) represents the phenotypic variance explained by the QTL; *Add* represents the additive effect. Positive values indicate a positive effect on SDS-SV of the ND3331 alleles, whereas negative values indicate a positive effect on SDS-SV of the Zang1817 alleles

**Fig. 1:**
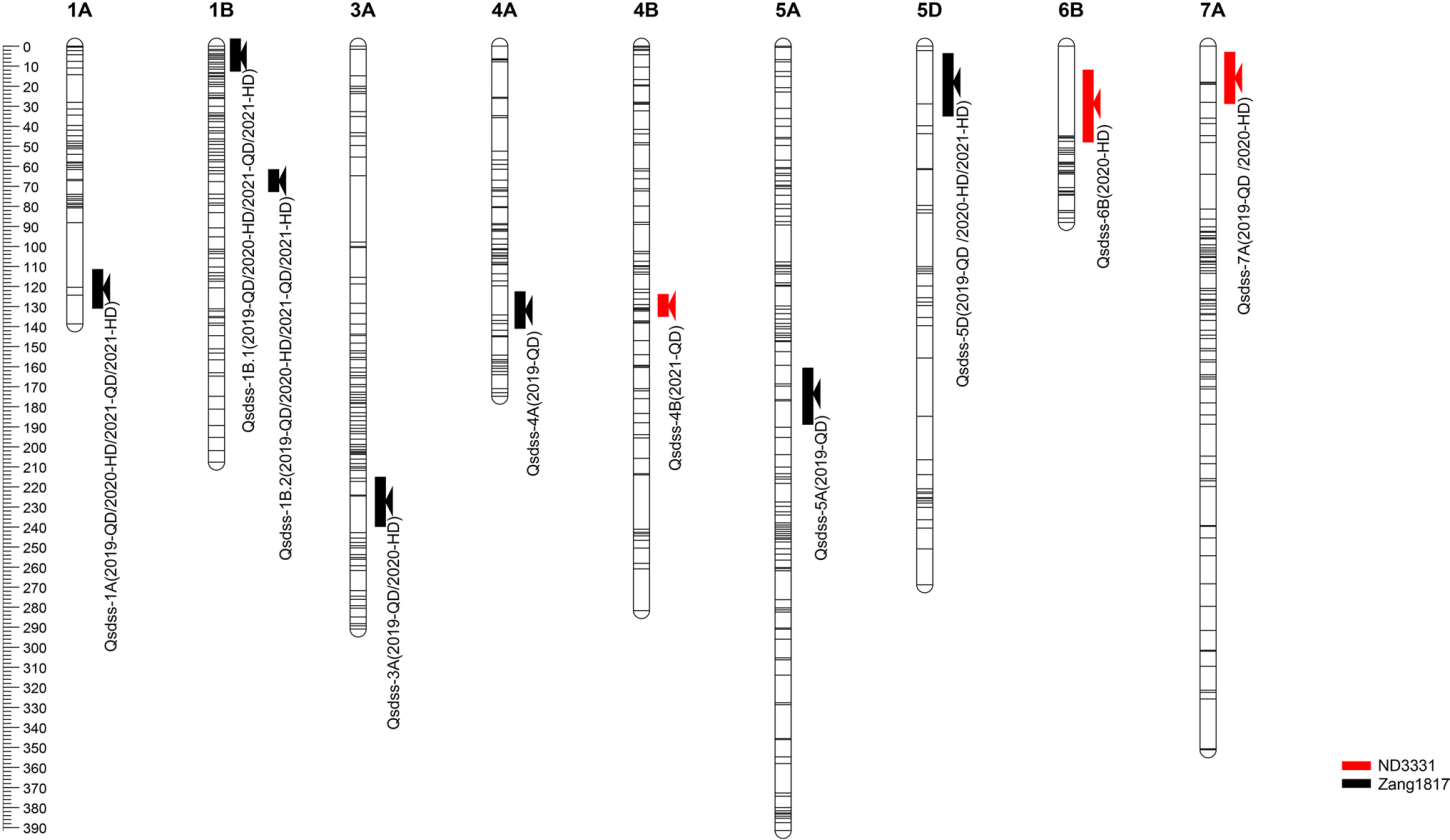
Distribution of quantitative trait loci (QTLs) identified in four environments. Map distances (cM) are indicated on the leaf of each chromosome, and marker names are on the right. The limit-of-detection (LOD) peak of each QTL is indicated by a bar; red bars indicate ND3331 alleles, and black bars indicate Zang1817 alleles. Genetic linkage maps were constructed using the software JoinMap 4.0 and MapChart (color figure online)

**Fig. 2:**
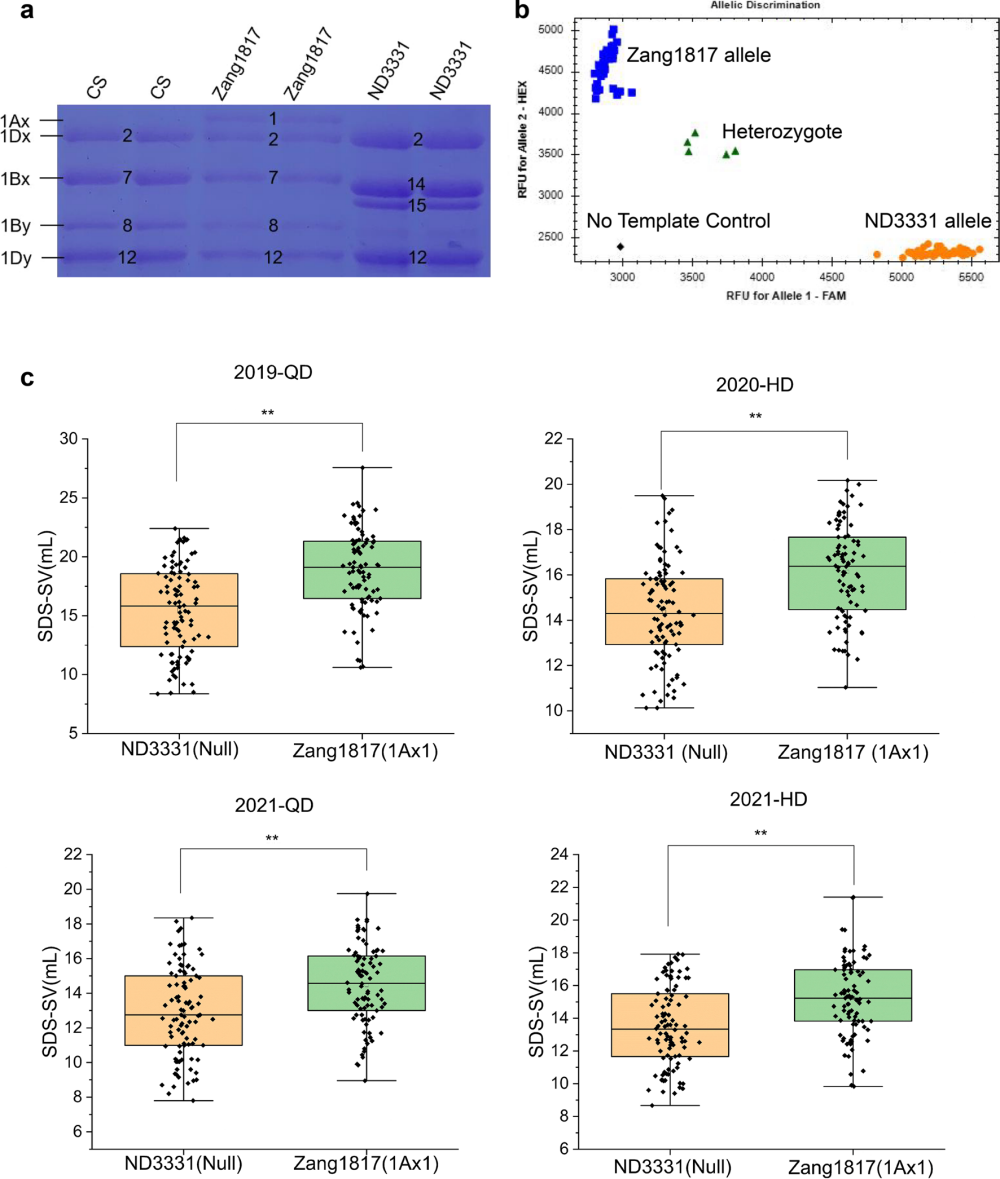
Effect of the 1Ax1 subunit at the *Glu-A1* locus on the SDS-SV. (**a**) High-molecular-weight glutenin subunit (HMW-GS) composition of ND3331 and Zang1817 detected by sodium dodecyl sulfate–polyacrylamide gel electrophoresis (SDS-PAGE), followed by Coomassie blue staining (CBS). Whole wheat flour (50 mg) was used for each sample. The wheat cultivar Chinese Spring (CS) (Null, 1Bx7 + 1By8, and 1Dx2 + 1Dy12) was used as the control. (**b**)The performance was described for 1Ax1 subunit linked single-nucleotide polymorphism markers in the RIL population. The blue and orange dots indicate RIL lines that have the same target marker genotype as in Zang1817 and ND3331, respectively; the green dots indicate heterozygotes, and the black dots indicate no template control. (**c**) The phenotypic effect of the 1Ax1 subunit in the 189 RILs according to the means of SDS-SV trait of different types. Double asterisks indicate significant differences determined by a two-tailed Student’s *t* test at *P* < 0.01 (color figure online)

The QTL *Qsdss-1A*, flanked by markers *AX-111601840* and *AX-110430171* on chromosome 1A, was stably detected at LOD ≥ 3.3 in four environments; the positive allele of this QTL was provided by Zang1817 and explained 4.09–9.21% of the phenotypic variances in SDS-SV (Table 3; Fig. 1 and Fig. S2). The physical interval flanked by these markers is 474.7–511.8 Mb of chromosome 1A, which contains the HMW-GS locus *Glu-A1* (at 508.7 Mb, IWGSC RefSeqv1.0). *Glu-A1* has been reported to explain 10.6% of the phenotypic variance in SDS-SV (Würschum et al. 2016). Sodium dodecyl sulfate–polyacrylamide gel electrophoresis (SDS-PAGE)(Chen et al. 2022) was used to determine the HMW-GS composition of ND3331 and Zang1817 and showed that Zang1817 contained the 1Ax1 subunit, which was absent in ND3331 (1Ax-Null) (Fig. 2a). We developed a kompetitive allele specific PCR (KASP) marker based on the SNP of the 1Ax subunit between parents ND3331 and Zang1817 (Dataset S1), and KASP analysis using this marker showed that the calls for the two alleles were clearly separated, with calls for the ND3331 allele clustered near the *X*-axis, while those for the Zang1817 allele were clustered near the *Y*-axis (Fig. 2b). These clustering results clearly distinguished the two alleles; therefore, we used the KASP marker to genotype the 189 RILs (Fig. 2b). The average SDS-SV of lines carrying the Zang1817 allele was significantly higher than that of lines carrying the ND3331 allele in all four environments (Fig. 2c). These results confirmed that HMW-GS on chromosome 1A was the major contributor to *Qsdss-1A* and 1Ax1 contributed to good end-use quality, compared with 1Ax-Null.

**Fig. 3:**
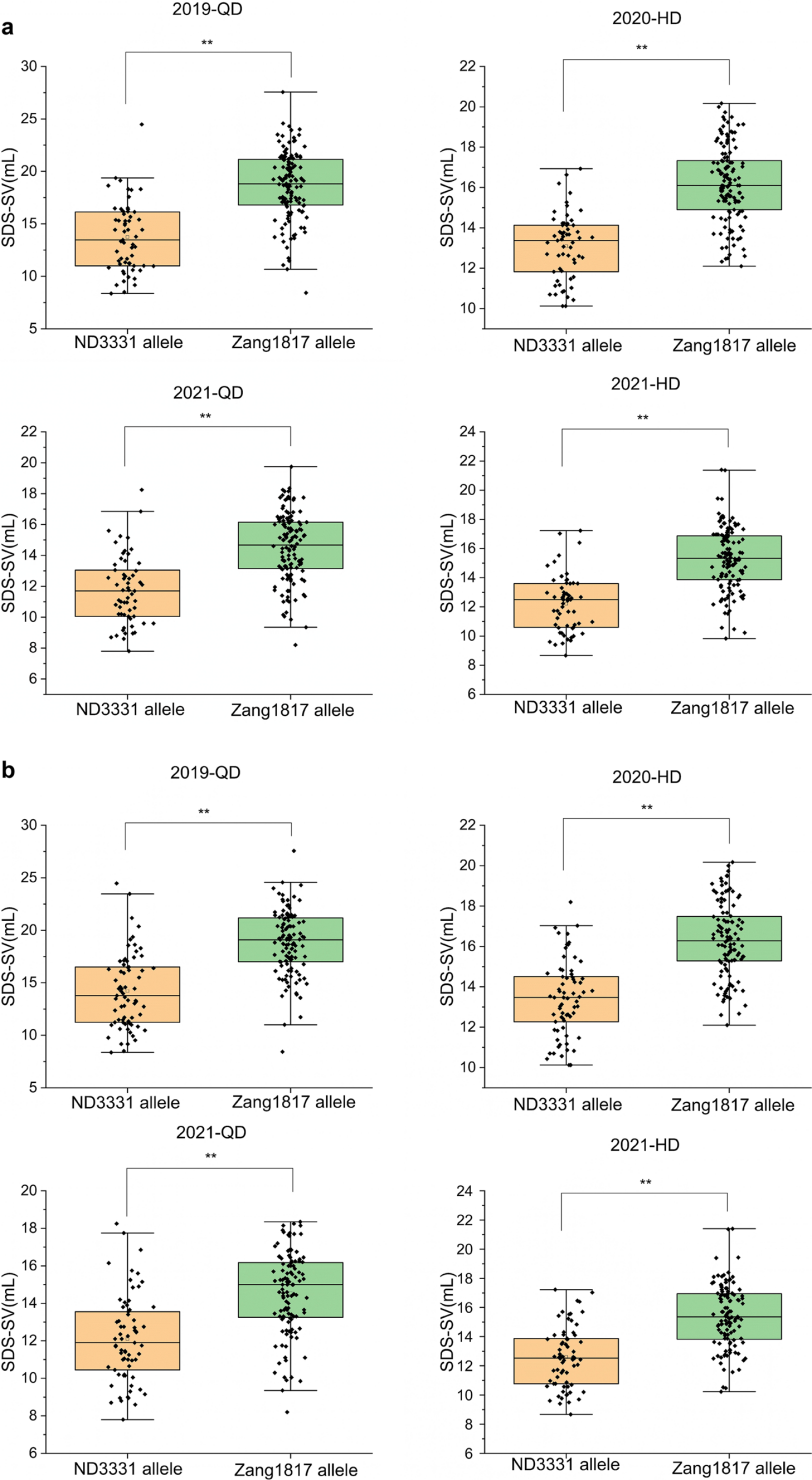
The phenotypic effect of 1BL/1RS translocation and *Glu-B1* locus in the 189 RILs according to the means of SDS-SV of different types. (**a**) 1BL/1RS translocation of ND3331 and Zang1817 are represented. (**b**) *Glu-B1* of ND3331 and Zang1817 are represented. Double asterisks indicate significant differences determined by a two-tailed Student’s *t* test at *P* < 0.01 (color figure online)

*Qsdss-1B.1*, located on chromosome 1B, was also stably identified at LOD ≥ 6.7 in four environments and was mapped to the intervals *AX-109503584*–*AX-108726602*, *AX-109840997*–*AX-108966369*, *AX-109840997*–*AX-108941599*, and *AX-109840997*–*AX-108941599*, depending on the environment. The positive allele of *Qsdss-1B.1* was provided by Zang1817 and accounted for 8.54–15.53% of the phenotypic variances in the SDS-SV trait (Table 3; Fig. 1 and Fig. S2). The physical interval of flanking markers was 1.3–347.6 Mb, which covered almost all of chromosome 1BS. Wheat chromosome 1BS carries the *Glu-B3* and *Gli-B1* loci, whereas rye chromosome 1RS carries the *Sec-1* locus, which encodes rye storage proteins (secalins) that affect bread-making quality (Howell et al. 2014). Previous studies indicated that the presence of 1B/1R translocation is associated with a serious quality defect, including low SDS-SV and reduced dough strength, and the negative effect of the 1B/1R translocation on SDS-SV is probably related to the loss of the *Glu-B3* and *Gli-B1* loci (Li et al. 2009; Würschum et al. 2016). Next, we determined whether ND3331 is a wheat-rye 1BL/1RS chromosome translocation line with replacement of wheat chromosome 1BS by rye chromosome 1RS. We used one pair of primers (ω-sec-F1/R1) designed from the rye ω-secalin gene (GenBank: FJ561476.1) on 1RS for genomic analysis (Dataset S1), and the results showed that a 0.95-kb fragment can be amplified from ND3331, while no fragment was amplified from Zang1817 (Fig. S3a). Thus, we speculated that the 1BL/1RS translocation might be the cause for the effect of *Qsdss-1B.1* on SDS-SV. We genotyped the 189 RILs using the codominant markers *ω-sec*-F1/R1, and *Glu-B3*-F1/R1 that designed from *Glu-B3* locus (GenBank: EU189089.1) on wheat chromosome 1BS, and the results revealed that RILs with ND3331 allele showed a 0.95 kb fragment and those with Zang1817 allele showed a 1.7 kb fragment (Fig. S3a, Dataset S1). The average SDS-SVs of lines with the Zang1817 allele were significantly higher than those of lines with the ND3331 allele (Fig. 3a). This result suggests that the effect of *Qsdss-1B.1* on SDS-SV is due to the 1BL/1RS translocation and more generally that the 1BL/1RS translocation is associated with inferior wheat quality.

*Qsdss-1B.2*, located on chromosome 1B, was consistently detected at LOD ≥ 8.1 in all environments. The left markers were all *AX-86184300*, while the right markers were *AX-111619113*, *AX-110041248*, *AX-111619113*, and *AX-110409210* in 2019-QD, 2020-HD, 2021-HD, and 2021-QD, respectively. *Qsdss-1B.2* explained 8.83–10.21% of the variation in SDS-SV in these environments (Table 3; Fig. 1 and Fig. S2). The physical interval of flanking markers was 555.3–577.7 Mb of chromosome 1B, which contains the HMW-GS locus *Glu-B1* located at 555.8 Mb (IWGSC RefSeqv1.0), suggesting that *Qsdss-1B.2* is likely *Glu-B1*. Moreover, ND3331 (Null, 1Bx14 + 1By15, 1Dx2 + 1Dy12) and Zang1817 (1, 1Bx7 + 1By8, 1Dx2 + 1Dy12) have different HMW-GS compositions at the *Glu-B1* locus (Fig. 2a). Thus, 1Bx7 + 1By8 from Zang1817 was associated with higher SDS-SV compared to 1Bx14 + 1By15 from ND3331. We again used the SNP variation at *Glu-B1* between the parents ND3331 and Zang1817 to develop a KASP marker (Dataset S1), and KASP analysis using this marker revealed that the calls for the two alleles were clearly separated, with those for the ND3331 allele clustered near the X-axis and those for the Zang1817 allele clustered near the Y-axis (Fig. S3b). We then analyzed the genotypes of the 189 RILs (Fig. S3b). The average SDS-SV of the lines with the Zang1817 allele was significantly higher than that of the lines with the ND3331 allele in all four environments (Fig. 3b). These results indicated that *Qsdss-1B.2* is mainly due to *Glu-B1*, and HMW-GS 1Bx7 + 1By8 makes a greater contribution to SDS-SV than 1Bx14 + 1By15.

The QTL *Qsdss-5D* in the interval *INDEL1489469* to *INDEL8181683* on chromosome 5D at LOD ≥ 5.2 was identified in three environments (2019-QD, 2020-HD, and 2021-HD), where it explained 5.71–10.80% of the phenotypic variances (Table 3; Fig. 1 and Fig. S2). The physical interval of flanking markers was 1.5–8.2 Mb, and this interval contains the grain hardness loci *Puroindoline a-D1* (*Pina-D1*) and *Puroindoline b-D1* (*Pinb-D1*) (located at 3.6 Mb, IWGSC RefSeqv1.0). SDS-SV is affected by the allelic variation at the *Pinb-D1* locus on chromosome 5DS (Ahn et al. 2014; Mohler et al. 2012; Würschum et al. 2016). It has been reported that *Pinb-D1* explains 4.7% of the genotypic variance in SDS-SV and that the *Pinb-D1b* allele has a positive effect on this trait (Würschum et al. 2016). Thus, we speculated that the effect of *Qsdss-5D* on SDS-SV may be due to the *Pinb-D1* locus.

To determine the effects of different genotype groups—*Glu-A1*, 1B/1R translocation, *Glu-B1*, and *Pinb-D1*, corresponding to *Qsdss-1A*, *Qsdss-1B.1*, *Qsdss-1B.2*, and *Qsdss-5D*, respectively, on SDS-SV trait, we developed polymorphic markers based on the variation between parents ND3331 and Zang1817. Then, we grouped the 189 RILs based on the allele at each locus: (a) ND3331 alleles and (b) Zang1817 alleles. This resulted in 16 different groups, in which we analyzed the SDS-SV trait (Table 4). The b-b-b-b group had the highest average SDS-SV (21.38 mL), followed by groups with three and then two b alleles, while the a-a-a-a group had the lowest SDS-SV (11.33 mL). These results indicate that the four loci have an additive effect on SDS-SV, with the Zang1817 alleles positively affecting wheat quality.

**Table 4:** SDS-SV of 189 RILs carrying different genotypes

Allele				SDS-SV (mL)
*Glu-A1*	1B/1R translocation	*Glu-B1*	*Pinb-D1*	
a	a	a	a	11.33d
a	a	a	b	12.49 cd
a	a	b	a	14.17bcd
a	b	a	a	14.37bcd
b	a	a	a	16.02bcd
b	a	b	a	16.08bcd
b	a	a	b	16.43abcd
a	a	b	b	16.75abc
a	b	a	b	16.78abc
b	b	a	a	16.97abc
a	b	b	a	17.27abc
b	a	b	b	15.67bcd
b	b	a	b	17.12abc
a	b	b	b	18.72ab
b	b	b	a	19.15ab
b	b	b	b	21.38a

*RIL* recombinant inbred line. *SDS-SV* sodium dodecyl sulfate-sedimentation volume. “a” and “b” indicate the ND3331 allele and the Zang1817 allele, respectively. *Glu-A1*, 1B/1R translocation, *Glu-B1*, and *Pinb-D1*, correspond to the QTLs *Qsdss-1A*, *Qsdss-1B.1*, *Qsdss-1B.2*, and *Qsdss-5D*, respectively. Values followed by the same letter are not significantly different at *P* < 0.05

## *Pinb-D1p* increases SDS-SV

To determine the effect of *Pin-D1* on the SDS-SV trait in the RIL population, we analyzed the full open reading frames (ORFs) of the *Pina-D1* and *Pinb-D1* alleles of the two parents, ND3331 and Zang1817. The results indicated that ND3331 contains the wild-type alleles *Pina-D1* and *Pinb-D1* (Fig. S4a and b; Fig. S5a and b), which have been previously defined as *Pina-D1a* and *Pinb-D1a* alleles, respectively (Giroux and Morris 1997, 1998)*.* However, ND3331 seeds are hard. To examine this further, we sequenced the promoter region of *Pina-D1a* of ND3331. *Pina-Pro-F/R* primers, located upstream of the translational start site, amplified a 2-kb band in CS and Zang1817 but not in ND3331 (Fig. S4c). We speculate that the promoter region of *Pina-D1a* in ND3331 contains a large insertion or deletion that could not be amplified; thus, we named this allele *Pina-D1a’*. An ORF sequence analysis of *Pina-D1* and *Pinb-D1* in Zang1817 revealed that it contains the wild-type *Pina-D1a* allele along with a mutated *Pinb-D1* allele with a one-base deletion at position 213 bp compared to *Pinb-D1a*, resulting in frameshift mutations leading to premature termination (Fig. S4a and b; Fig. S5a and b). This mutated *Pinb-D1* allele was previously named *Pinb-D1p* (Bhave and Morris 2008).

**Fig. 4:**
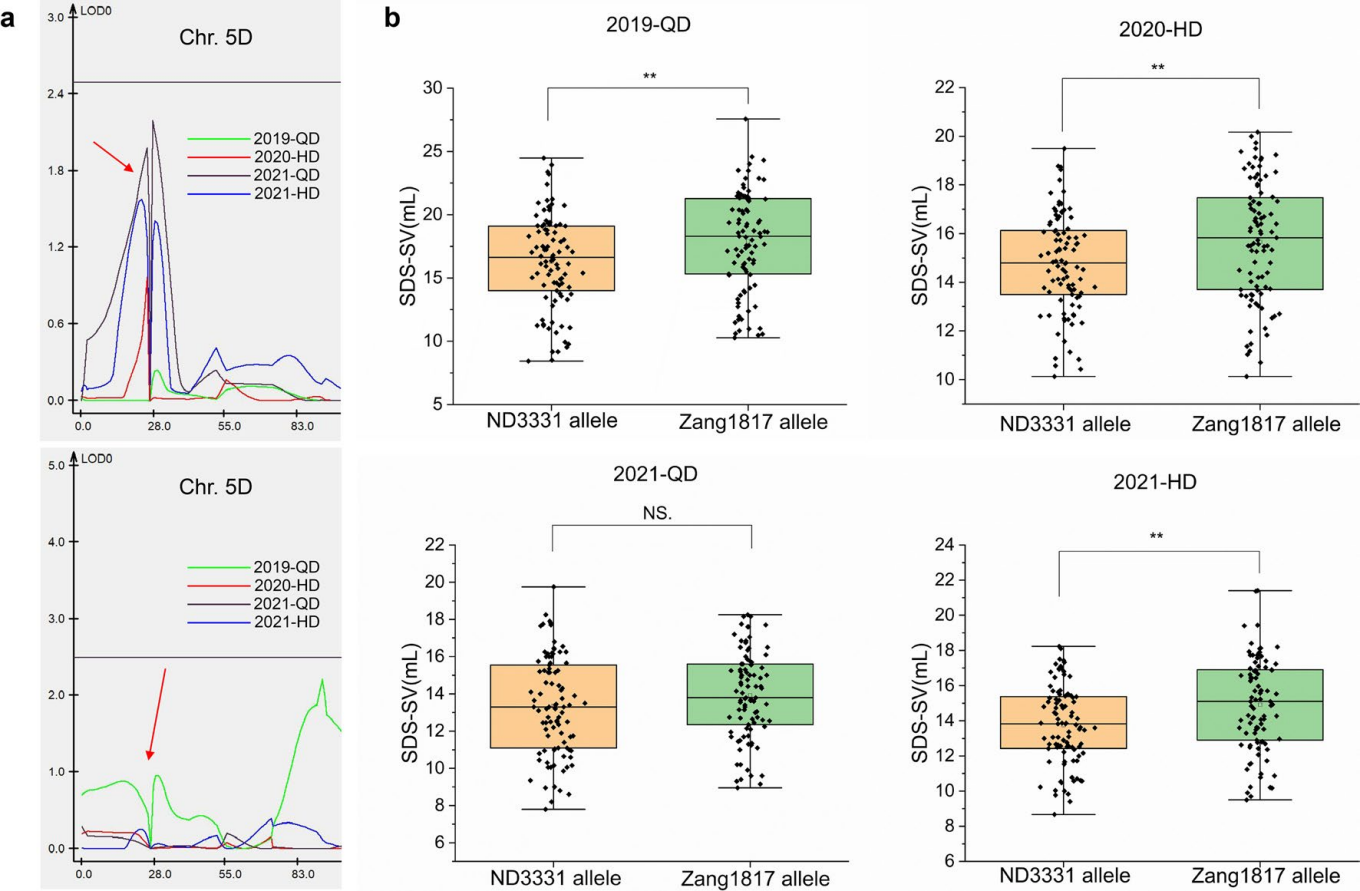
The effects of *Pina-D1* and *Pinb-D1* alleles on SDS-SV in the RIL population. (**a**) The effect of QTL *Qsdss-5D* on SDS-SV in the two groups divided by *Pina-D1*/*Pinb-D1* alleles in the RILs. The red arrows indicate the *Qsdss-5D*. (**b**) Boxplots showing the SDS-SV of genotypes carrying different *Pina-D1*/*Pinb-D1* alleles in four environments. Double asterisks indicate significant differences determined by a two-tailed Student’s *t* test at *P* < 0.01 (color figure online)

We developed a KASP marker based on the one-base deletion in the *Pinb-D1a* allele in ND3331 and the *Pinb-D1p* allele in Zang1817 (Dataset S1), and KASP analysis using this marker revealed that the calls for the two alleles were clearly separated, with those for the ND3331 allele clustered near the X-axis and those for the Zang1817 allele clustered near the Y-axis (Fig. S3c). We used the KASP marker to genotype the 189 RILs, and two groups were obtained, one containing *Pina-D1a’/Pinb-D1a* (ND3331 alleles) and the other containing *Pina-D1a/Pinb-D1p* (Zang1817 alleles) (Fig. S3c). In each group, the effect of *Qsdss-5D* on SDS-SV was eliminated with LOD < 2.5 (Fig. 4a), indicating that *Pina-D1/Pinb-D1* might be the major contributor to *Qsdss-5D*. Moreover, the group with Zang1817 alleles had a significantly higher average SDS-SV than the group with ND3331 alleles in three environments (2019-QD, 2020-HD, and 2021-HD) (Fig. 4b), which indicates that the *Pina-D1a/Pinb-D1p* alleles have a positive effect on SDS-SV compared with the *Pina-D1a’/Pinb-D1a* alleles. Together, these results suggest that *Qsdss-5D* is likely due to the *Pin* locus, and the additive effect of the Zang1817 allele on SDS-SV was due to the *Pina-D1a/Pinb-D1p* genotype.

**Fig. 5:**
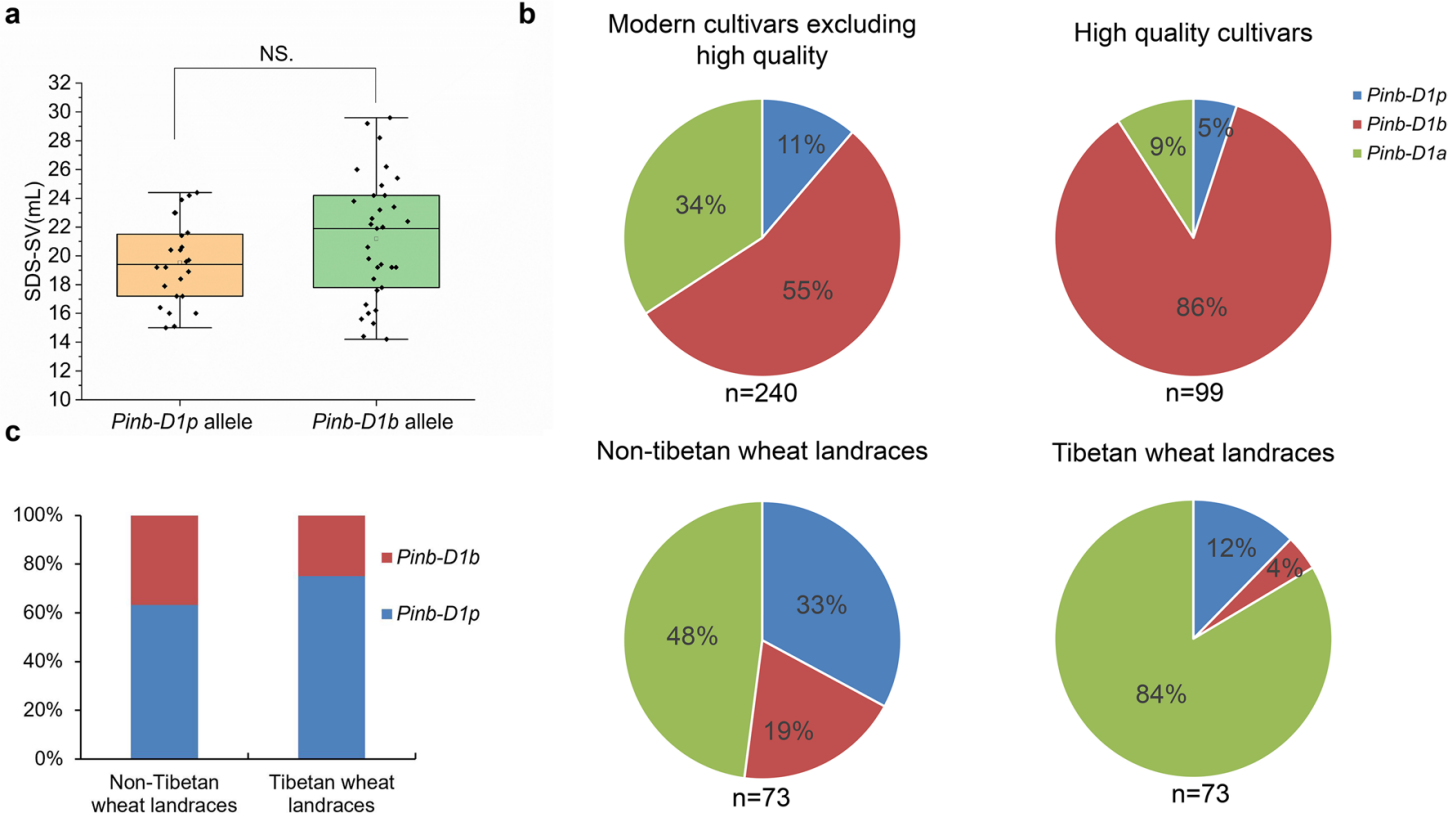
Comparison of the effects of *Pinb-D1b* and *Pinb-D1p* alleles on SDS-SV and the distribution frequencies of the *Pinb-D1a*, *Pinb-D1p*, and *Pinb-D1b* alleles among different wheat materials. (**a**) Comparison of the effects of *Pinb-D1b* and *Pinb-D1p* alleles on SDS-SV. A total of 24 samples with the *Pinb-D1p* allele and 33 samples with the *Pinb-D1b* allele were compared. NS indicates no significant difference. (**b**) The distribution frequencies of the *Pinb-D1a*, *Pinb-D1p*, and *Pinb-D1b* alleles in four groups of wheat materials, including modern cultivars (excluding high-quality cultivars), high-quality cultivars, landraces from non-Tibetan areas, and landraces from Tibet. (**c**) The distribution frequencies of the *Pinb-D1a* and *Pinb-D1p* alleles for non-Tibetan landraces and Tibetan wheat landraces (color figure online)

It has been reported that the *Pinb-D1b* allele also have a positive effect on SDS-SV (Würschum et al. 2016). To compare the effects of the *Pinb-D1b* and *Pinb-D1p* alleles on SDS-SV, we genotyped 485 wheat materials including modern wheat cultivars and Chinese landrace accessions for *Pinb-D1* alleles and found that 65 of the materials contained the *Pinb-D1p* allele and 233 contained the *Pinb-D1b* allele (Dataset S3). Since HMW-GS has a great effect on the SDS-SV trait, we examined the HMW-GS composition of these 298 materials using SDS-PAGE and then screened 57 materials containing identical HMW-GSs (Null, 1Bx7 + 1By8/1By9, and 1Dx2 + 1Dy12) for SDS-SV analysis. The average SDS-SV of the 24 materials with the *Pinb-D1p* allele (19.5 mL) was lower than that of the 33 materials with the *Pinb-D1b* allele (21.1 mL), but there was no significant difference between the two groups (Fig. 5a, Dataset S4). These results indicate that the *Pinb-D1p* allele may have a similar positive effect as *Pinb-D1b* on the SDS-SV trait.

We analyzed the distribution frequencies of the *Pinb-D1a*, *Pinb-D1p*, and *Pinb-D1b* alleles for four groups of wheat: high-quality cultivars, modern cultivars (excluding high-quality cultivars), landraces from non-Tibetan areas, and landraces from Tibet (Fig. 5b, Dataset S3). *Pinb-D1b* was the most common *Pinb-D1* allele in the modern wheat cultivars and high-quality cultivars, with a frequency of 55% and 86%, respectively (Fig. 5b), indicating that selection for the *Pinb-D1b* allele from landraces occurred during wheat quality improvement. The *Pinb-D1p* allele had a low frequency, at 11% in modern cultivars and 5% in high-quality cultivars. However, *Pinb-D1p* was more common in landraces from non-Tibetan areas and Tibet, with frequencies of 33% and 12%, respectively (Fig. 5b), suggesting that *Pinb-D1p* might be an elite allele that could be utilized in wheat quality improvement in addition to *Pinb-D1b*. Moreover, the *Pinb-D1p*/*Pinb-D1b* ratio in Tibetan wheat landraces was significantly higher than that in non-Tibetan landraces (Fig. 5c), suggesting, more generally, that landraces from Tibet areas may contain underutilized elite genotypes that could be used in wheat quality improvement.

## Discussion

The SDS-sedimentation volume (SDS-SV) is one of the important wheat quality parameters related to gluten quality and quantity, and high SDS-SV has been associated with stronger gluten and superior bread-making quality. In the present study, we identified 10 QTLs for the SDS-SV using the ND3331/Zang1817 RIL population, which have been mapped to 1A, 1B, 3A, 4A, 4B, 5A, 5D, 6B and 7A chromosomes. Among them, four major QTLs including *Qsdss-1A*, *Qsdss-1B.1*, *Qsdss-1B.2*, and *Qsdss-5D*, were identified and their effects on SDS-SV were due to the *Glu-A1* locus encoding the high-molecular-weight glutenin subunit 1Ax1, the 1B/1R translocation, 1Bx7 + 1By8 at the *Glu-B1* locus, and the hardness-controlling loci *Pina-D1* and *Pinb-D1*, respectively.

High-molecular-weight glutenin subunits were highly associated with SDS-SV. Previous reports showed that many QTLs associated with SDS-SV was mapped on chromosome 1A (Chen et al. 2015,2020; Deng et al. 2015; Li et al. 2009; Semagn et al. 2021; Tian et al. 2021; Würschum et al. 2016; Yang et al. 2020: Yu et al. 2017), and these QTLs were mainly affected by allelic variations at *Glu-A1* (508.7 Mb, RefSeq v1.0) and *Glu-A3* (4.2 Mb, RefSeq v1.0) loci (Deng et al. 2015; Li et al. 2009; Semagn et al. 2021; Würschum et al. 2016). *Glu-A1* contributed 10.6% of the phenotypic variance of SDS-SV (Würschum et al. 2016). A major effect QTL *QSv.dms-1A* at 1.3 Mb on chromosome 1AS explaining up to 37% of the phenotypic variation were likely either *Glu-A3* or *Gli-A3* (Semagn et al. 2021). Our research confirmed that *Glu-A1* located at 474.7–511.8 Mb on chromosome 1AL associated with the SDS-SV in four environments and we provided the genetic evidence that HMW-GS 1Ax1 contributed to better end-use quality compared with 1Ax-null. *Qsdss-1B.2* located at 555.3–577.7 Mb on chromosome 1BL is mainly due to the HMW-GS locus *Glu-B1* (555.8 Mb, IWGSC RefSeq v1.0), which is consistent with previous studies showing that QTLs for SDS-SV on chromosome 1B were mainly associated with *Glu-B1* (Chen et al. 2020; Conti et al. 2011; Huang et al. 2006; Patil et al. 2009; Semagn et al. 2021; Würschum et al. 2016). However, these studies have not investigated the effects of different HMW-GS allelic variations at *Glu-B1* on SDS-SV trait. Our study provides genetic evidence that HMW-GS 1Bx7 + 1By8 makes a greater contribution to SDS-SV than 1Bx14 + 1By15.

Grain hardness is one of the most important characteristics for milling and baking quality of wheat (Pasha et al. 2010), and the grain hardness index is positively correlated with SDS-SV (Shang et al. 2021). Previous studies revealed that SDS-SV is affected by the allelic variation at the *Pinb-D1* locus on chromosome 5DS (Ahn et al. 2014; Mohler et al. 2012; Würschum et al. 2016). It has also been reported that *Pinb-D1* explains 4.7% of the genotypic variance in SDS-SV and that the *Pinb-D1b* allele has a positive effect on this trait (Würschum et al. 2016). In this study, we identified a stable QTL *Qsdss-5D* located at 1.5–8.2 Mb on chromosome 5DS associated with the SDS-SV and indicated that the grain hardness loci *Pina-D1* and *Pinb-D1* (located at 3.6 Mb, RefSeqv1.0) is the major contributor to *Qsdss-5D*. More importantly, we showed for the first time that *Pinb-D1p* allele have a positive effect on SDS-SV, and the effect of which on the SDS-SV trait may similar to *Pinb-D1b*. We also analyzed the distribution frequencies of different *Pinb-D1* alleles for four groups of wheat, and found that *Pinb-D1b* allele has been utilized in wheat quality improvement. However, *Pinb-D1p* allele had a low frequency in modern cultivars, especially in high-quality cultivars, suggesting that *Pinb-D1p* might be an elite allele that could be utilized in wheat quality improvement in addition to *Pinb-D1b*. Moreover, we found a higher *Pinb-D1p*/*Pinb-D1b* ratio in landraces from Tibet areas, where high-altitude conditions trigger extensive reshaping of wheat genomes that providing a significant resource for wheat genetic improvement (Guo et al. 2020a), than that in non-Tibetan landraces, suggesting that landraces from Tibet areas may contain underutilized elite genotypes that could be used in wheat quality improvement.

In summary, this study identified ten QTLs associated with the SDS-SV trait in wheat using an RIL population derived from a hybrid between the common wheat line ND3331 and the Tibetan semi-wild wheat accession Zang1817. It provides genetic evidence that 1Bx7 + 1By8 makes a greater contribution to SDS-SV than 1Bx14 + 1By15. Importantly, the *Pinb-D1p* allele had a positive effect on SDS-SV and therefore could be used by breeders to aggregate high-quality genes for wheat quality improvement. The molecular markers developed in this study will be helpful for selecting breeding lines with high SDS-SV and gluten strength. These results lay important foundation for future investigation into potential candidate genes. Further research using this population will provide much-needed insights and knowledge about SDS-SV, which is an important quality trait.

## Supplementary Information

Below is the link to the electronic supplementary material.

Supplementary file1 (DOCX 1381 kb)

Supplementary file2 (XLSX 12 kb)

Supplementary file3 (XLSX 16 kb)

Supplementary file4 (XLSX 21 kb)

Supplementary file5 (XLSX 12 kb)

## Data Availability

The genomic sequencing data have been deposited to NCBI Sequence Read Archive with accession number PRJNA596843 (Guo et al. 2020a).
