# Supplementary material for: Pinb-D1p is an elite allele for improving end-use quality in wheat (Triticum aestivum L.)
Source: Theor Appl Genet. 2022 Sep 29;135(12):4469–81. doi: 10.1007/s00122-022-04232-7 (PMC9734229; doi:10.1007/s00122-022-04232-7)
Supplement: Supplementary file 1 — Supplementary file1 (DOCX 1381 kb) [file 122_2022_4232_MOESM1_ESM.docx]

**Supplementary information**


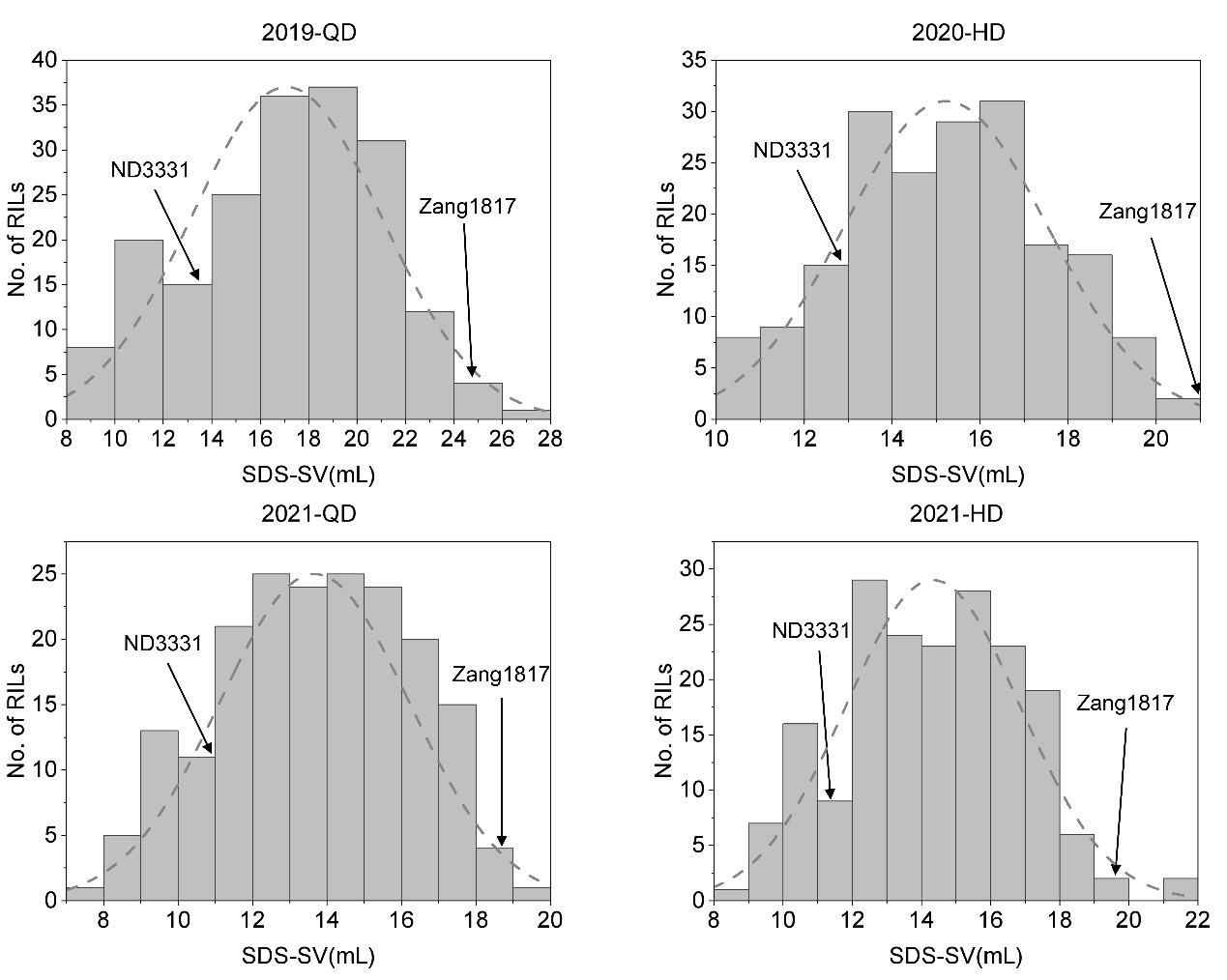


**Fig. S1** **Histograms of the sodium dodecyl sulfate-sedimentation volume (SDS-SV) in an ND3331 and Zang1817 recombinant inbred population.** The four environments were Qingdao in 2019 (2019-QD), Handan in 2020 (2020-HD), Qingdao in 2021 (2021-QD), and Handan in 2021 (2021-HD). The Y-axis represents the number of RILs of the SDS-SV trait of different group spacing, and the X-axis represents the phenotypic data. The arrow indicates the phenotypic range of the two parents.


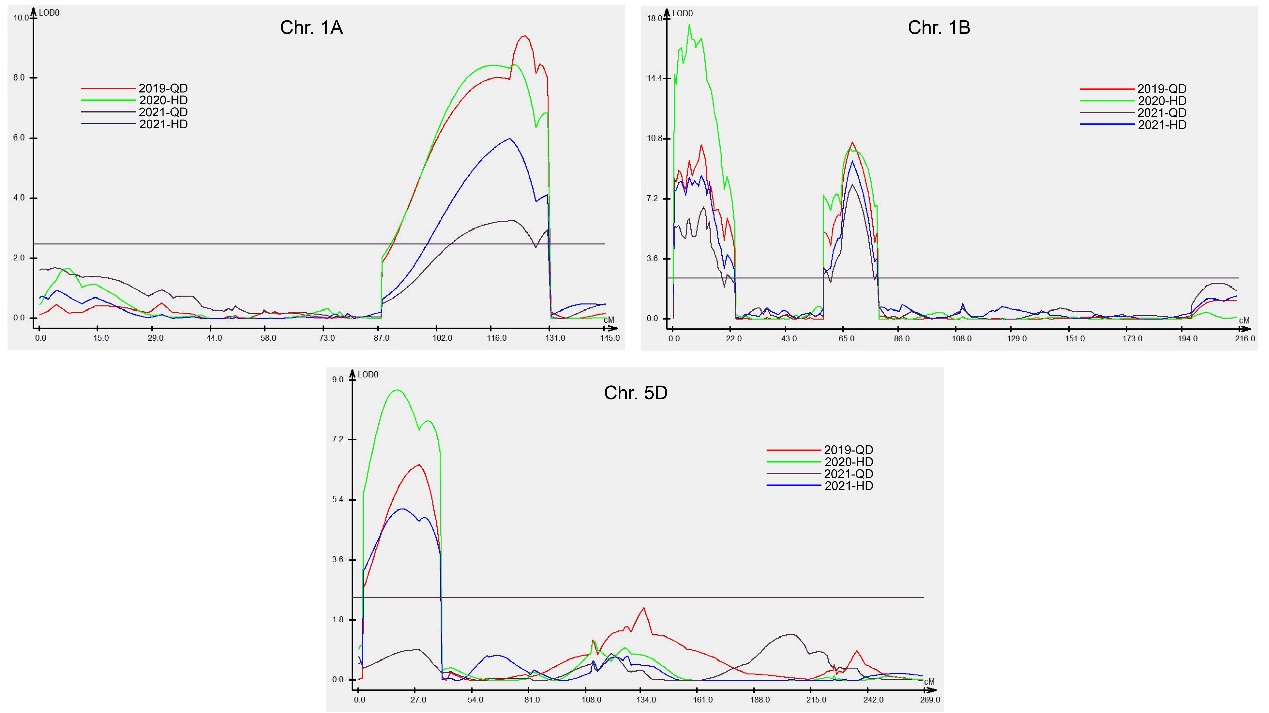


**Fig. S2** **QTL analysis of sodium dodecyl sulfate-sedimentation volume (SDS-SV) in four environments.** The four environments were Qingdao in 2019 (2019-QD), Handan in 2020 (2020-HD), Qingdao in 2021 (2021-QD), and Handan in 2021 (2021-HD).


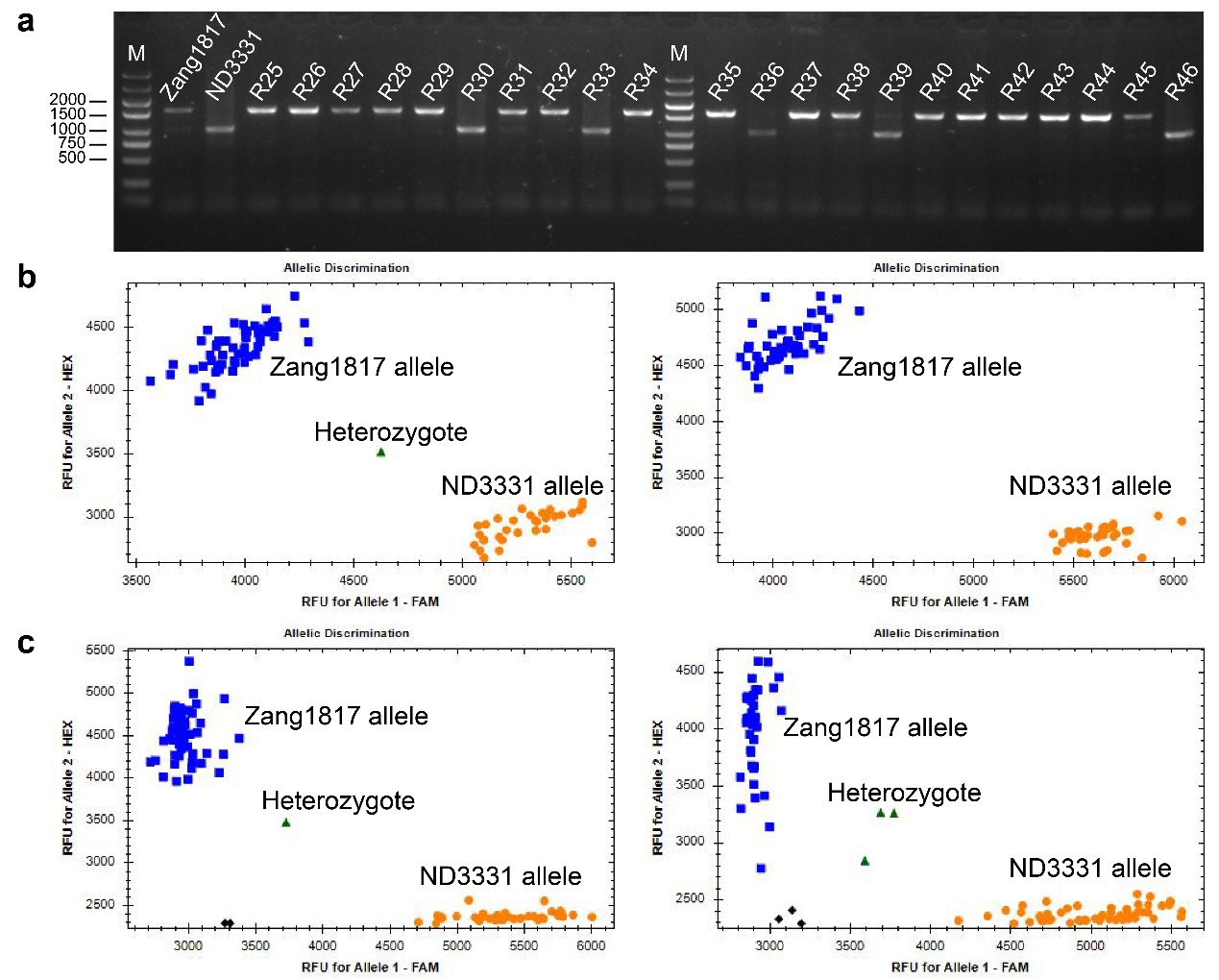


**Fig. S3** **Performance of the (a) 1BL/1RS translocation, (b) *Glu-B1*, and (c) *Pinb-D1* allele linked polymorphism markers in the two parents and the RIL population. (a)** Agarose gel images of the two parents and partial RIL lines. M, DNA marker. The sizes of the bands in ND3331 and Zang1817 were 0.95 kb and 1.7 kb, respectively. The blue and orange dots in **(b)** and **(c)** indicate RIL lines that have the same target marker genotype as in Zang1817 and ND3331, respectively; the green dots indicate heterozygotes, and the black dots indicate no template control.


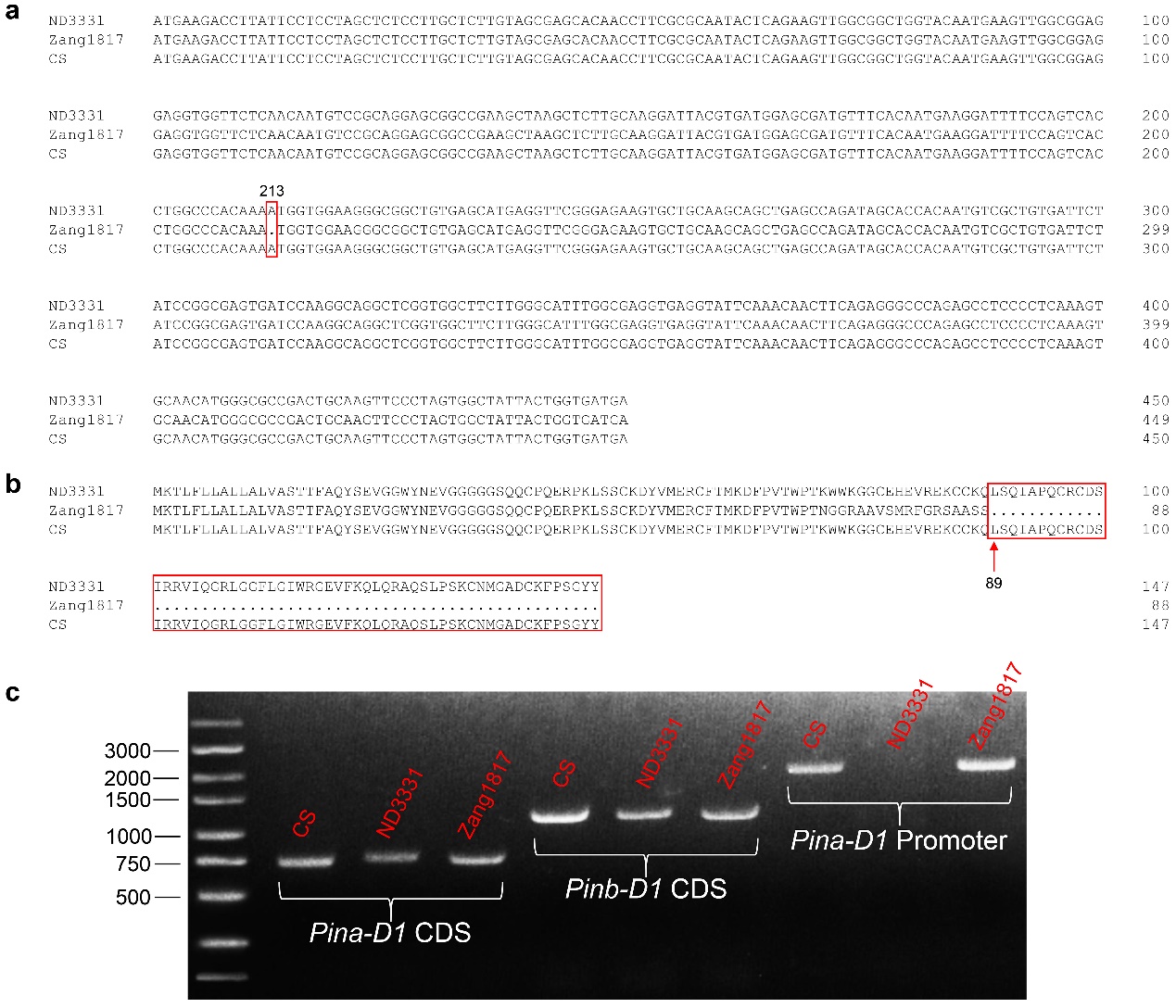


**Fig. S4** **Alignments of the coding sequences and deduced amino acid sequences of *Pinb-D1* in Nongda3331 and Zang1817. (a)** Coding sequence alignments of *Pinb-D1* between ND3331 and Zang1817. The wheat cultivar Chinese Spring (CS) with the wild-type *Pinb-D1a* allele was used as the control. The red frame highlights the single base deletion in Zang1817. **(b)** Amino acid sequence alignments of *Pinb-D1* between ND3331 and Zang1817. The wheat cultivar Chinese Spring (CS) with the wild-type *Pinb-D1a* allele was used as the control. The red frame highlights the variation. **(c)** Amplification of *Pina-D1* and *Pinb-D1* coding sequences and *Pina-D1* promoter regions from CS, ND3331, and Zang1817.


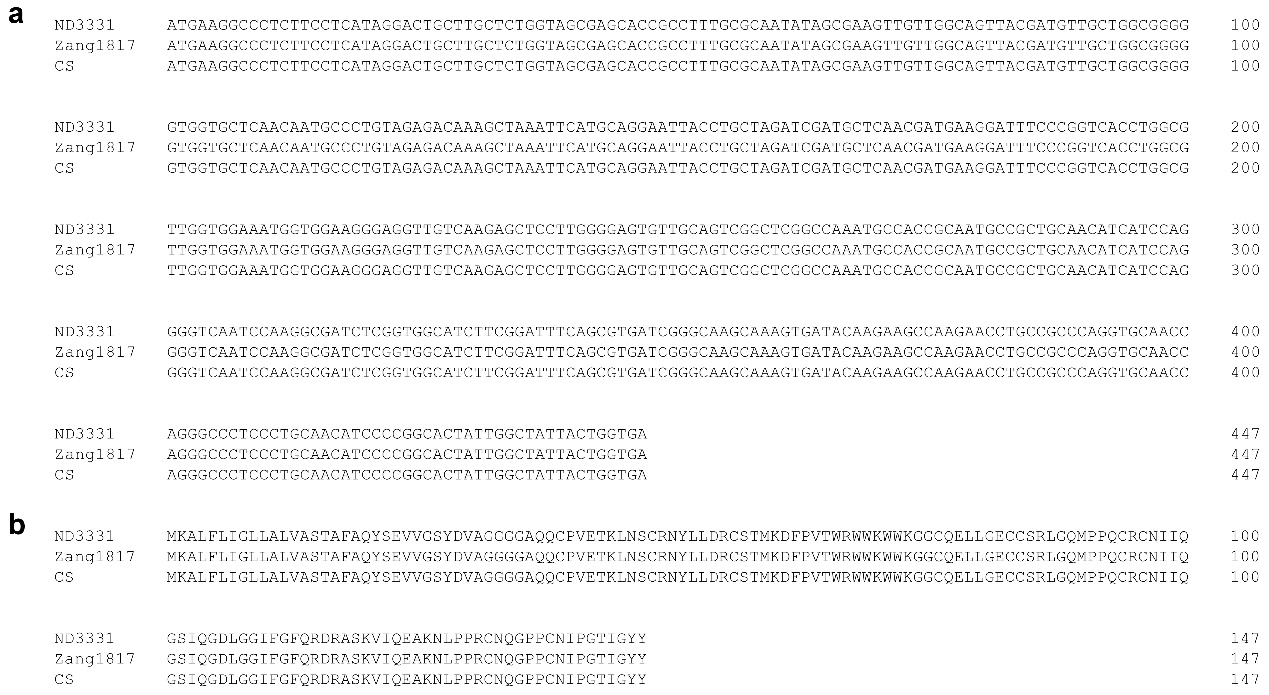


**Fig. S5 Alignments of the coding sequences and deduced amino acid sequences of *Pina-D1* in Nongda3331 and Zang1817. (a)** Coding sequence alignments of *Pina-D1* between ND3331 and Zang1817. The wheat cultivar Chinese Spring (CS) with the wild-type *Pina-D1a* allele was used as the control. **(b)** Amino acid sequence alignments of *Pina-D1* between ND3331 and Zang1817. The wheat cultivar Chinese Spring (CS) with the wild-type *Pina-D1a* allele was used as the control.

**Table S1** **Statistics of the constructed genetic map using the recombinant inbred line (RIL) population.**

| Chromosome | No. of linkage groups | No. of markers | Length (cM) | Average locus interval (cM) |
| --- | --- | --- | --- | --- |
| 1A | 1 | 39 | 123.95 | 3.18 |
| 1B | 1 | 80 | 207.63 | 2.60 |
| 1D | 1 | 56 | 221.66 | 3.96 |
| 2A | 1 | 51 | 332.05 | 6.51 |
| 2B | 1 | 70 | 399.95 | 5.71 |
| 2D | 1 | 14 | 50.8 | 3.63 |
| 3A | 1 | 85 | 290.95 | 3.42 |
| 3B | 1 | 56 | 237.89 | 4.25 |
| 3D | 1 | 23 | 217.03 | 9.44 |
| 4A | 1 | 56 | 174.65 | 3.12 |
| 4B | 1 | 68 | 281.72 | 4.14 |
| 4D | 1 | 40 | 150.81 | 3.77 |
| 5A | 1 | 110 | 391.44 | 3.56 |
| 5B | 1 | 87 | 495.1 | 5.69 |
| 5D | 1 | 34 | 265.59 | 7.81 |
| 6A | 1 | 33 | 168.74 | 5.11 |
| 6B | 1 | 26 | 88.02 | 3.39 |
| 6D | 1 | 53 | 333.5 | 6.29 |
| 7A | 1 | 77 | 351.24 | 4.56 |
| 7B | 1 | 32 | 260.55 | 8.14 |
| 7D | 1 | 84 | 465.57 | 5.54 |
| A | 7 | 451 | 1,833.02 | 4.06 |
| B | 7 | 419 | 1,970.86 | 4.70 |
| D | 7 | 304 | 1,704.96 | 5.61 |
| Total | 21 | 1,174 | 5,508.84 | 4.69 |
